# Employing an Incentive Spirometer to Calibrate Tidal Volumes Estimated from a Smartphone Camera

**DOI:** 10.3390/s16030397

**Published:** 2016-03-18

**Authors:** Bersain A. Reyes, Natasa Reljin, Youngsun Kong, Yunyoung Nam, Sangho Ha, Ki H. Chon

**Affiliations:** 1 Department of Biomedical Engineering, University of Connecticut, Storrs, CT 06269, USA; bersain.reyes@engineer.uconn.edu (B.A.R.); reljin@engr.uconn.edu (N.R.); 2 Department of Computer Science and Engineering, Soonchunhyang University, Asan 336-745, Korea; vengeful224@gmail.com (Y.K.); yynams@gmail.com (Y.N.); hsh.oopsla@gmail.com (S.H.)

**Keywords:** tidal volume, incentive spirometer, smartphone camera, calibration, breathing monitor

## Abstract

A smartphone-based tidal volume (V_T_) estimator was recently introduced by our research group, where an Android application provides a chest movement signal whose peak-to-peak amplitude is highly correlated with reference V_T_ measured by a spirometer. We found a Normalized Root Mean Squared Error (NRMSE) of 14.998% ± 5.171% (mean ± SD) when the smartphone measures were calibrated using spirometer data. However, the availability of a spirometer device for calibration is not realistic outside clinical or research environments. In order to be used by the general population on a daily basis, a simple calibration procedure not relying on specialized devices is required. In this study, we propose taking advantage of the linear correlation between smartphone measurements and V_T_ to obtain a calibration model using information computed while the subject breathes through a commercially-available incentive spirometer (IS). Experiments were performed on twelve (*N* = 12) healthy subjects. In addition to corroborating findings from our previous study using a spirometer for calibration, we found that the calibration procedure using an IS resulted in a fixed bias of −0.051 L and a RMSE of 0.189 ± 0.074 L corresponding to 18.559% ± 6.579% when normalized. Although it has a small underestimation and slightly increased error, the proposed calibration procedure using an IS has the advantages of being simple, fast, and affordable. This study supports the feasibility of developing a portable smartphone-based breathing status monitor that provides information about breathing depth, in addition to the more commonly estimated respiratory rate, on a daily basis.

## 1. Introduction

Tidal volume (V_T_) provides information about the breathing depth and is defined as the volume of air moved with each breath. Normal average V_T_ is approximately 0.5 L but this volume varies as the mechanism of respiratory control adjusts both V_T_ and respiratory rate (RR) in response to different activities, for example exercise or sleep, to meet the body’s requirements [1]. Tidal volume is important information to measure during mechanical ventilation to ensure sufficient ventilation without trauma to the lungs. Moreover, for patients with chronic obstructive pulmonary diseases, having the luxury to estimate their tidal volume at homes could be beneficial. For example, upon asthma attack, having not only the respiratory rate but also the tidal volume of the patient would give a physician better quantification of the severity of the asthma attack.

Several clinical and research methods currently exist to estimate V_T_ including spirometry, impedance pneumography, inductance plethysmography, photoplethysmography, Doppler radar, computed tomography, phonospirometry, and electrocardiography [2,3,4,5,6,7,8,9,10]. However, these devices have largely been designed for clinical or research centers and hence they are not applicable for everyday use for home monitoring due to the complexity of the devices, their high cost, their need for skilled operators, and in some cases their limited portability.

An interesting approach to overcome some of the abovementioned limitations is to use general purpose video cameras to optically monitor breathing. Although most efforts in this area have focused on estimation of the RR [11,12,13,14,15,16], there have also been studies to estimate V_T_ [17,18,19,20]. Recently, a volume conservation hypothesis was proposed by establishing a one-to-one linear relationship between changes of the external torso volume and V_T_ corresponding to internal lung air content [6]. Previous findings also indicated that accurate V_T_ estimation results by tracking markers placed on the chest wall surface via an optical reflectance system [17,18]. Although promising, these approaches are difficult to apply to the general population outside research setting do the reasons listed above. More recently, a good correlation (*r*^2^ = 0.81) between shoulder displacements obtained by processing webcam video recordings and exhaled breath volume measured with a commercial metabolic analysis device was obtained [19]. Besides the promising results, analysis in terms of V_T_ estimation was limited to the correlation between the video amplitudes and reference volumes. In addition, the implementation was done in a personal computer and with the aid of an external digital camera.

Smartphones’ fast microprocessors, multiple sensors, large data storage, software flexibility, and media capabilities are attractive for developing monitoring systems that can potentially be used by the general public. They have accordingly been found to be accurate in a diversity of vital sign monitoring applications [21,22,23,24]. Recently, our research group proposed an approach using dual cameras consisting of contact and noncontact video monitoring directly implemented on a smartphone for estimation of heart rate (HR) and the mean RR, respectively [16]. In that study, noncontact video monitoring of the chest area provided waveforms whose amplitudes were concordant with either the increase or decrease in the depth of breathing. In a subsequent study, we analyzed use of the non-contact approach for the task of V_T_ estimation and tracking of RR at each time instant [20]. We found that the peak-to-peak amplitude of the smartphone-acquired chest movement signal was highly correlated with the V_T_ from the spirometer, which was regarded as the reference (*r*^2^ = 0.951 ± 0.042, mean ± SD). We found that when calibrated on an individual basis, the root mean squared error (RMSE) was 0.182 ± 0.107 L, which is equivalent to 14.998% ± 5.171% when normalized (NRMSE). Besides this former effort, we are not aware of a smartphone-based system for V_T_ estimation using an optical approach together with an algorithm directly implemented on a smartphone. However, we recognize that in order to be used outside research and clinical settings by the general population, a simple calibration procedure that does not employ an expensive, complicated and specialized device is required.

In this paper we propose a calibration procedure using a volume-oriented incentive spirometer (IS) for the task of V_T_ estimation from the smartphone-acquired chest movement signal. To this end, we computed the V_T_ estimates after calibration from data computed while breathing through an IS, and compared them to simultaneously-acquired volume from a spirometer as reference. The performance of the V_T_ estimation from the proposed calibration via IS was also compared to the best estimation that could be obtained via linear regression between the reference volume and smartphone data. The smartphone application used in this study was implemented in a commercially-available Android smartphone and its screens used for V_T_ estimation are described in this paper.

## 2. Material and Methods

### 2.1. Subjects

Twelve (*N* = 12) healthy and non-smoker volunteers (eleven males) aged 27.7 ± 9.5 years (mean ± standard deviation), weight 71.6 ± 7.8 kg, and height 174.5 ± 6.0 cm, were recruited for this study. Individuals with previous pneumothorax, those with chronic respiratory illnesses such as asthma, and anyone who had symptoms of the common cold or an upper respiratory infection were excluded from this study. Each volunteer consented to be a subject and signed the study protocol approved by the Institutional Review Board of the University of Connecticut (UConn, Storrs, CT, USA).

### 2.2. Signal Acquisition

*Equipment*. The algorithm for recording the chest movement signal was implemented in an HTC One M8 smartphone (HTC Corporation, New Taipei City, Taiwan) running the Android v4.4.2 operating system. The frontal camera of this smartphone was used, which had a 5 MP, backside-illumination sensor with wide angle lens and 1080 p full HD video recording capabilities at 30 frames-per-second. The implemented application processed the video data in real time to obtain the chest movement signal for estimation of V_T_. Collected data were saved into a text file for offline analysis of results in Matlab (R2012a, The Mathworks, Natick, MA, USA).

To test the smartphone-based V_T_ estimates, a reference volume signal was collected with a spirometer system consisting of a respiration flow head connected to a differential pressure transducer for measuring the airflow signal (MLT1000L, FE141 Spirometer, ADInstruments, Dunedin, New Zealand). The integral of the airflow over time was computed to generate the volume signal. Both the airflow and volume signals were sampled at 1 kHz using a 16-bit A/D converter (PowerLab/4SP, ADInstruments, Sydney, Australia). Prior to recording, the spirometer system was calibrated using a 3.0 L calibration syringe (Hans Rudolph, Inc., Shawnee, KS, USA). Each volunteer was provided with a new breathing apparatus set consisting of a disposable filter, mouthpiece, and nose clip (MLA304, MLA1026, MLA1008, ADInstruments). For calibration of the smartphone-based V_T_ estimates, a new volumetric incentive spirometer (IS) was provided to each volunteer (Airlife^TM^, Carefusion, Yorba Linda, CA, USA).

*Breathing maneuvers*. Each experiment consisted of two phases with the corresponding maneuvers as follows:

- *Calibration maneuver*. Volunteers were asked to breathe four times through the IS, inhaling to a first target of 250 mL, then hold their breath for 2 s, and finally breathe four times through the IS to a second inhalation target of 500 mL.
- *Test breathing maneuver*. Volunteers were asked to hold their breath for 2 s, take a deep breath, and then breathe through the spirometer system to different inhalation volume levels ranging from around 200 mL to 2.5 L; first increasing their V_T_ with each inhalation for around one minute, and finally decreasing their V_T_ with each inhalation for another minute. Subjects breathed at their own pace, *i.e.*, we did not use a metronome to control their respiratory frequency. Reference volume was recorded for this maneuver using spirometry.

**Figure 1:**
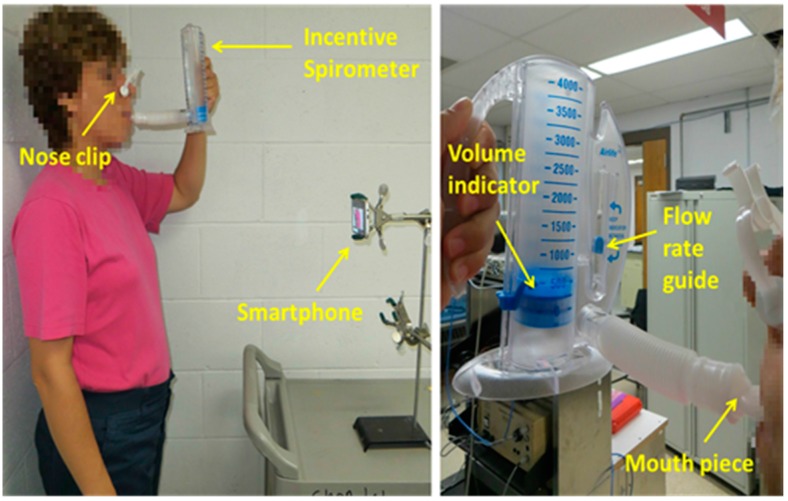
Experimental setup to record the chest movements using the smartphone camera while volunteers breathe through an incentive spirometer (IS) for calibration. (**Left**) General view of the experimental setup of the calibration maneuver; (**Right**) Detailed view of the IS while the subject is inspiring to reach a volume target. Subjects were asked to inspire so that the top of the piston lined up with the desired blue mark and at a rate that kept the indictor between the two blue guide arrows.

Data from the calibration maneuver was used to compute the calibration model for the smartphone-based V_T_ estimates. As seen in Figure 1, the IS we used has increments of 250 mL, a volume indicator, and a flow rate guide. Volunteers were asked to hold the IS in its upright position and then breathe through the mouthpiece of the IS so that at each inspiration the top of the volume indicator lined up with the corresponding target mark, while the flow rate indicator was kept in between the two arrow guides to maintain an adequate inspiration speed as indicated in the manufacturer’s manual. While the volunteers performed the calibration maneuver, the chest movement signal was recorded using the smartphone placed in front of the subject at approximately 60 cm in a 3-pronged clamp at their thoracic level. It is worth mentioning that the volume signal from the spirometer was not recorded during the calibration maneuver as the volunteers were breathing through the IS mouthpiece. Hence, the exact volume inspired at each breathing phase of the calibration maneuver was not collected, but fixed at the predefined target levels. Before starting the calibration maneuver, the volunteers learned how to use the IS and were allowed to practice and familiarize themselves with the maneuver. The smartphone application we developed allows a remote Start/Stop recording option via a generic Bluetooth^®^ camera shutter (I Shutter, Shanghai, China).

The second (test) maneuver provided a wide range of V_T_ to test the computed calibration model. Simultaneous recording of the smartphone-based chest movement signal and spirometer-acquired reference volume was performed. The chest movement signal was recorded in the same manner as it had been for the calibration maneuver. Visual feedback was provided to the volunteers by displaying the reference volume on a 40″ monitor placed in front of them, where visual marks were used to indicate the tidal volume’s range of interest.

Both maneuvers were recorded in a regular dry lab using ambient light from ceiling fluorescent lamps. During both maneuvers, subjects were asked to stand still and not to change position in between maneuvers. Nose clips were used to clamp the nostrils during both maneuvers. A concern that arises when employing a non-contact optical approach for breathing monitoring is the ability of the system to capture the breathing-related movements when the subjects are wearing different colors and patterns, as this approach looks for changes in the light intensity due to the modification of the path length caused by breathing displacements of the chest wall. Hence, during the experiments, volunteers had the freedom to wear different colored and patterned clothes like plain or stripes, and were only asked not to wear loose clothes. As with other breathing monitoring techniques, e.g., inductance plethysmography, the quality of the signal and ultimately the performance of the monitoring system could degrade if clothes are too loose to see chest movements. An example of the acquisition setup is shown in Figure 1 for a subject breathing through the IS.

### 2.3. Smartphone Algorithm for Recording Chest Movements

The chest movement signal *I*(*t*) was computed in real time in the smartphone app by averaging the intensity within a rectangular region of interest (ROI) of the red, green and blue (RGB) channels at each time instant *t*, according to

(1) $$I\left(t\right)=\left(\frac{1}{3D}\right)\left(\underset{\left\{m,n\right\}\in ROI}{\sum }i_{R}\left(m,n,t\right)+\underset{\left\{m,n\right\}\in ROI}{\sum }i_{G}\left(m,n,t\right)+\underset{\left\{m,n\right\}\in ROI}{\sum }i_{B}\left(m,n,t\right)\right)$$

where *i_x_(m,n,t)* is the intensity value of the pixel at the *m*-th row and *n*-th column of the RGB channel within the ROI containing a total of *D* pixels. The camera resolution was set to 320 × 240 pixels, and the ROI of 49 × 90 pixels was focused on the thoracic area of the volunteer. The sampling rate fluctuated around 25 frames-per-second during the real time monitoring. Hence, after stopping the recording, the recorded signal was cubic splined to obtain a uniform sampling rate of 25 Hz. Finally, a bandpass filter was applied to the chest movement signal between 0.01 and 2 Hz using a 50th order finite impulse response (FIR) filter, designed with a Hamming window, to minimize the high frequency components not related to the breathing maneuvers and the trend in the signal. Both the cubic spline and bandpass filtering were performed in the smartphone app. The conditioned signals of the maneuvers were saved in a text file for further analysis in a personal computer.

### 2.4. Data Preprocessing

The reference volume signal recorded during the second phase of the experiment (the test maneuver) was analyzed offline in Matlab. First, it was down-sampled to 25 Hz to achieve the same sampling frequency as the corresponding chest movement signal, and then bandpass filtered using a 4th-order Butterworth bandpass between 0.01 and 2 Hz applied in a forward and backward scheme to produce zero-phase distortion and minimize the start and end transients.

**Figure 2:**
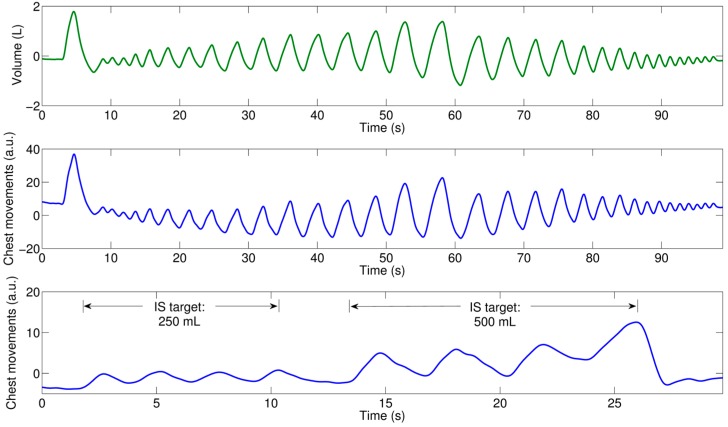
Example of acquired signals for the two breathing maneuvers of a subject: (**Top**) reference volume from spirometer for the test maneuver; (**Middle**) corresponding chest movement signal for the test maneuver recorded via the proposed smartphone camera app. These two signals from test maneuver were aligned due to different starting times; and (**Bottom**) chest movement signal recorded during calibration maneuver while the subject was breathing though the incentive spirometer; four inspirations at 250 mL target, and four inspirations at 500 mL target. Inspirations/expirations correspond to positive/negative deflections in the signals.

Due to differences in the starting times and delays between the smartphone and spirometer systems, simultaneously recorded signals were aligned using the initial breath holding and deep inspiration portion of the data and also by using the cross-correlation function, where 20 s in the central portion of the maneuver were extracted from each recording to compute the cross-correlation sequence and find the sample lag with the maximum cross-correlation value indicating the required samples to be shifted. Figure 2 shows an example of the acquired signals using smartphone and spirometer, after alignment, for the maneuvers performed by one subject.

### 2.5. Calibration Using Incentive Spirometer

The inspiratory segments of the calibration maneuver using IS were identified from the chest movement signal. Information from the expiratory segments of the calibration maneuver was not used, as the volume indicator of the IS returns to its original position mainly due to gravity and not by the expiratory effort of the volunteer. Then, the peak-to-peak amplitude of this signal was computed at each inspiration and matched with the corresponding target volume from the IS. This resulted in two data sets: (1) four data points with ordinate values $V_{IS,1}$ at 250 mL; and (2) four data points with ordinate values $V_{IS,2}$ at 500 mL, with each point having an abscissa Δx equal to the peak-to-peak amplitude of the chest movement signal for that corresponding inspiratory phase.

Next, the median peak-to-peak amplitude of each set was computed so that the information from the IS maneuver was condensed into two data points, *A* and *B*, as follows:

(2) $$\begin{array}{c} A=\left(\hat{\Delta x_{1}},V_{IS,1}\right)=\left(\hat{\Delta x_{1}},250\;mL\right)\\ B=\left(\hat{\Delta x_{2}},V_{IS,2}\right)=\left(\hat{\Delta x_{2}},500\;mL\right) \end{array}$$

where $\hat{\Delta x_{1}}$ and $\hat{\Delta x_{2}}$ are the median values of the peak-to-peak amplitudes of the chest movement signal for the inspirations at 250 mL target ($V_{IS,1}$) and at the 500 mL target ($V_{IS,2}$), respectively. Finally, the calibration curve to map the peak-to-peak amplitudes to tidal volume estimates was obtained using the linear equation given the locations of points *A* and *B*:

(3) $$V_{Tsmartphone}=\left(\frac{V_{IS,2}-V_{IS,1}}{\hat{\Delta x_{2}}-\hat{\Delta x_{1}}}\right)\left(\Delta x-\hat{\Delta x_{1}}\right)+V_{IS,1}$$

which in turn can be written as

(4) $$V_{Tsmartphone}=\left(\frac{250}{\hat{\Delta x_{2}}-\hat{\Delta x_{1}}}\right)\Delta x+250\left(1+\frac{\hat{\Delta x_{1}}}{\hat{\Delta x_{2}}-\hat{\Delta x_{1}}}\right)$$

where $V_{Tsmartphone}$ denotes the tidal volume estimate given the peak-to-peak amplitude of the smartphone-acquired chest movement signal and the data from calibration using IS.

### 2.6. Tidal Volume Estimation Using Smartphone

After we applied the calibration linear model obtained from the calibration maneuver, the V_T_ smartphone estimates were tested, using the tidal volumes obtained from the spirometer as reference. To this end, the maxima and minima of the reference volumes were found and the $V_{Tspirometer}$ were computed as the absolute amplitude difference between two consecutive extrema. The corresponding peak-to-peak amplitudes Δx were found in the smartphone-acquired chest movement signals. Finally, the linear model obtained from the IS, given by Equation (4), was applied to each value Δx of the maneuver to obtain the corresponding smartphone-based V_T_ estimate.

The performance of the estimation was measured on the test data in terms of the root-mean-squared error *RMSE*, given by

(5) $$RMSE=\sqrt{\frac{\sum_{i=1}^{M}{\left(V_{T_{spirometer}}\left(i\right)-V_{T_{smartphone}}\left(i\right)\right)}^{2}}{M}}$$

and its normalized version *NRMSE* with respect to the mean tidal volume of the maneuver, given by

(6) $$NRMSE=\frac{RMSE}{mean\left(V_{T_{\text{spirometer}}}\right)}\;\times \;100\%$$

where $V_{T_{spirometer}}$ indicates the reference tidal volume measured by the spirometer, $V_{T_{smartphone}}$ the tidal volume estimated from smartphone-acquired chest movements after calibration with the IS model, and *M* is the number of breath-phases of the analyzed breathing maneuver.

In addition, these V_T_ estimates obtained from calibration via IS data were compared to those V_T_ obtained when applying a linear regression to the absolute peak-to-peak amplitude of the chest movement signal and the simultaneously-recorded reference V_T_ from the spirometer, to see how much the estimates from IS calibration deviate from those obtained with the best estimation model in the least-squares sense.

## 3. Results

**Figure 3:**
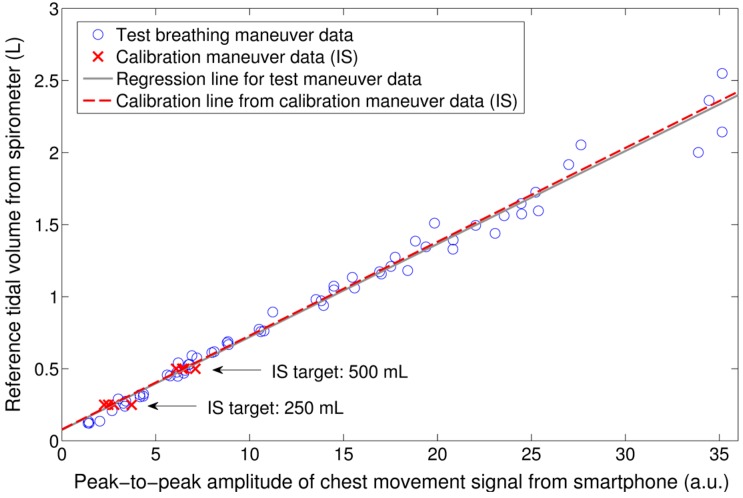
Example of simultaneously-acquired data using the smartphone camera and spirometer for one subject’s experiment. The solid gray line is the regression line for the test maneuver. Red crosses indicate the data collected during the test maneuver while the subject was breathing at 250 mL and 500 mL targets through the incentive spirometer (IS). The calibration model computed from the IS data is indicated by the red dashed line.

Reference tidal volumes from the spirometer distributed from a minimum of 0.190 ± 0.116 L (mean ± SD), to a maximum of 2.607 ± 0.400 L, with an average of 1.024 ± 0.159 L for the maneuvers performed by all volunteers (*N* = 12). As in our previous study [20], a strong linear correlation between the peak-to-peak amplitude of the chest movement signal from the smartphone’s camera and the reference V_T_ from the spirometer was found (*r*^2^ = 0.945 ± 0.037). An example of this relationship for the breathing maneuver of one subject is shown in Figure 3. The distribution of *r*^2^ values was not normal, as tested using a one-sample Kolmogorov-Smirnov test (*p* = 0.017). The median *r*^2^ was found to be higher than 0.9 as tested by a one-sample Wilcoxon signed rank test (*p* = 0.002). The RMSE and NRMSE errors obtained when mapping the peak-to-peak amplitude of the chest movement signal to V_T_ quantities using linear regression is shown in Table 1.

**Figure 4:**
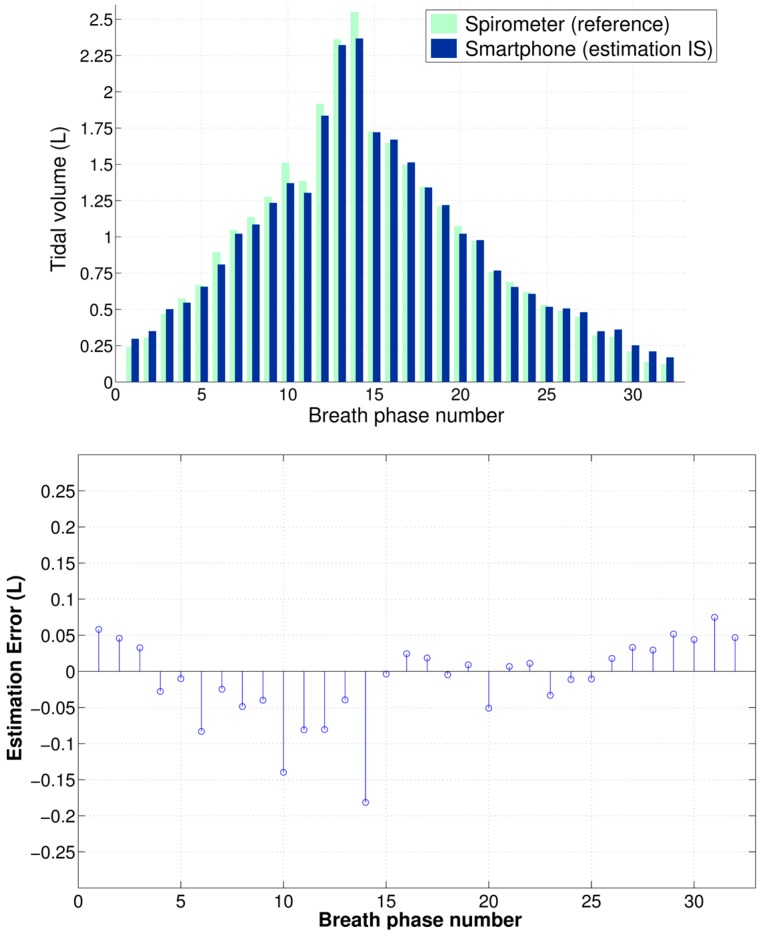
Example of tidal volume estimation using the smartphone-acquired chest movement signal calibrated with an incentive spirometer (IS) for the test maneuver performed by one volunteer. For visualization purposes, only data from inspiratory phases are displayed. (**Top**) Side-to-side tidal volumes; and (**Bottom**) corresponding estimation errors of smartphone-system with respect to spirometry.

To calibrate the peak-to-peak amplitude of the chest movement signal from the smartphone, two data points were extracted from the calibration maneuver using IS and a linear model was computed from these points to map the smartphone quantities to tidal volumes. An example of the data extracted from the calibration maneuver using IS and the corresponding calibration model are shown in Figure 3 together with the testing data from simultaneously-recorded V_T_ from the spirometer and peak-to-peak amplitude of the chest movement signal. Figure 4 shows an example of the V_T_ estimation using the smartphone data calibrated via the IS for the test maneuver of a subject as well as the corresponding estimation errors with respect to reference volume from spirometry. Table 1 shows the performance indices obtained for all subject for the V_T_ estimates from the smartphone when the proposed calibration via IS was used.

**Figure 5:**
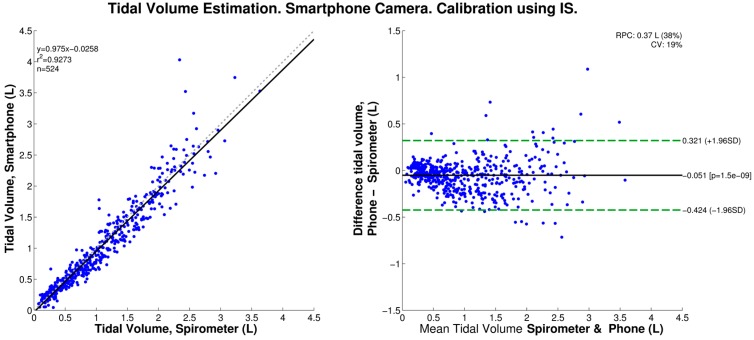
Tidal volume estimation from smartphone-acquired chest movement signal calibrated using incentive spirometer (IS) (*N* = 12 subjects). (**Left**) Regression curve: Grey dashed line indicates the identity line and the solid black the regression line; (**Right**) Bland-Altman plot: Solid black line indicates the bias and dashed green lines indicate the 95% limits of agreement.

**Figure 6:**
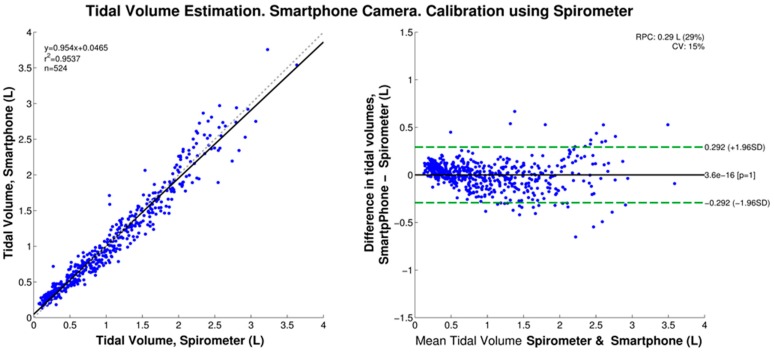
Tidal volume estimated via linear regression of smartphone-acquired data and reference tidal volume from spirometer (*N* = 12 subjects). (**Left**) Regression curve: Grey dashed line indicates the identity line and the solid black the regression line; (**Right**) Bland-Altman plot: Solid black line indicates the bias and dashed green lines indicate the 95% limits of agreement.

We found that, when calibrated using the IS data, the smartphone-based V_T_ estimation produced a statistically-significant bias of −0.051 L, and 95% limits of agreement of −0.424 and 0.321 L, as shown in the corresponding Bland-Altman plot of Figure 5. In contrast, when the peak-to-peak amplitudes were mapped to volumes using the linear regression of the simultaneously-acquired spirometer data, no statistically-significant bias was found, and the 95% limits of agreement were ±0.292 L as shown in Figure 6.

In our previous study using spirometer data for calibration, the RMSE and NRMSE values of the smartphone-based V_T_ estimates were found to be 0.182 ± 0.107 L and 14.998% ± 5.171%, respectively [20]. Those RMSE and NRMSE values did not distribute normally, as tested by a one-sample Kolmogorov–Smirnov test (*p* = 0.008 and *p* = 0.017, respectively). When comparing those prior results to those obtained in this study, using the best model from the regression of spirometer and smartphone data, no statistically-significant differences (*p* = 0.961) were found. Finally, the estimation errors obtained in this study from the calibration via IS were compared to those from the linear regression by means of a paired-sample *t*-test and statistically-significant increases in the mean value of the RMSE (*p* = 0.007) and NRMSE (*p* = 0.004) using IS were found.

**Figure 7:**
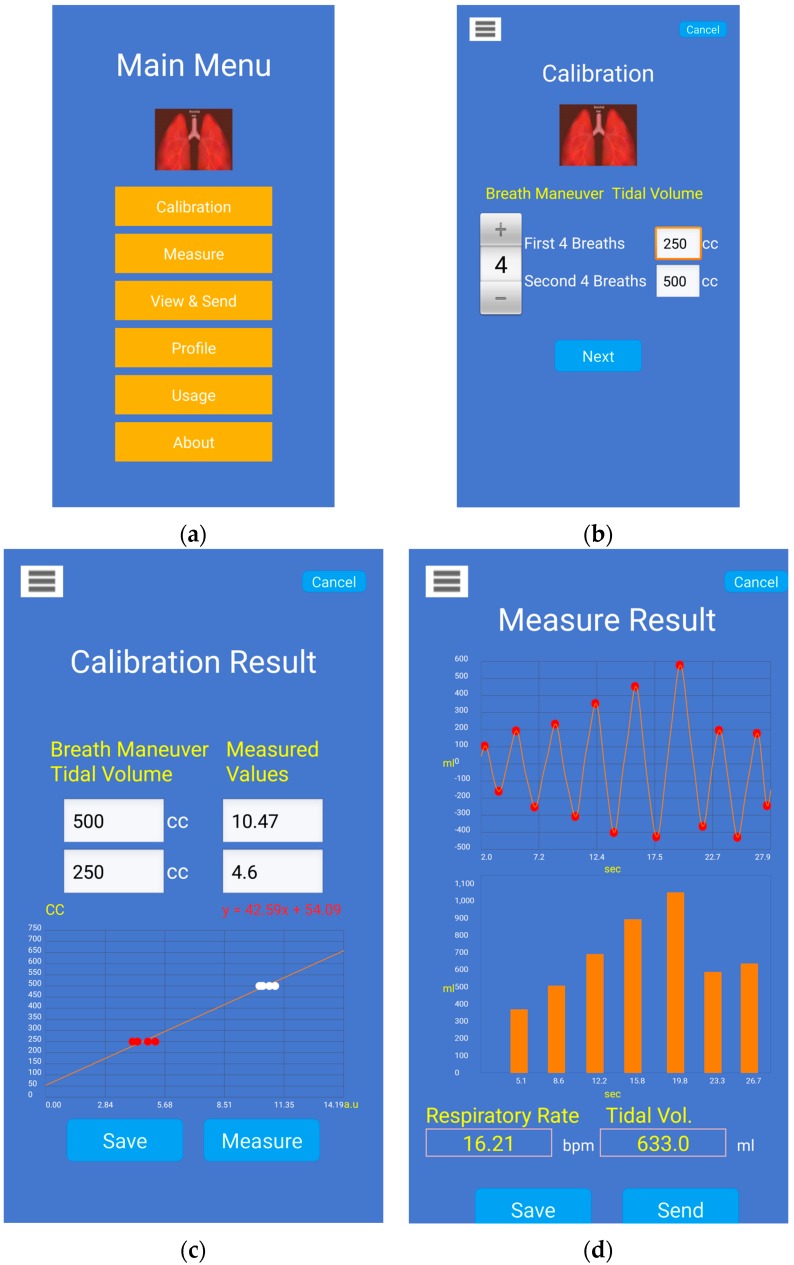
Screenshots of the Android smartphone application prototype for tidal volume estimation using the camera and calibration with an incentive spirometer (IS): (**a**) main menu of the Android application; (**b**) calibration setup, which allows adjusting the number of inspirations and target volumes; (**c**) example of calibration model computed while the subject breathed through the IS, where red dots indicate the first IS target (250 mL) and white dots the second target (500 mL); and (**d**) example of tidal volume estimates after calibration, where the top waveform indicates the chest movement signal from the smartphone camera, the bottom graph displays the estimated tidal volume of each inspiration and the average respiratory rate and tidal volume are also displayed.

Four screenshots of the smartphone app are shown in Figure 7 for the task of V_T_ estimation with calibration via IS. Figure 7a shows the main menu of the Android app. Figure 7b shows the settings screen for the calibration maneuver with IS which allows the user to adjust the number of breaths and corresponding target volumes in IS. Figure 7c shows an example of the calibration model computed from the breathing data through an IS. Once the calibration model is computed, it is stored for further measurement of V_T_. Figure 7d shows an example of calibrated V_T_ estimates from the smartphone’s chest movement signal, where the figure on top displays the processed waveform and detected breath phase onsets, and the figure at the bottom displays the corresponding V_T_ estimates during inspiratory phases. The average RR and average V_T_ are also displayed on this screen.

## 4. Discussion and Conclusions

This study is an extension of our previous work [20] that proposed the estimation of V_T_ directly on a smartphone by processing video recording information to obtain a chest movement signal correlated with reference V_T_ from a spirometer. Compared to our previous study [20], the novel aspects of this work include: (1) the introduction of an easy calibration procedure based just on the chest wall movement information recorded using the smartphone camera while breathing a few times through an inexpensive incentive spirometer device; and (2) the full implementation of the proposed signal processing algorithms on a smartphone app which makes it now possible for subjects to wirelessly control the calibration and measurement of tidal volume by themselves. Here, we innovated a simple and attainable calibration procedure to easily allow V_T_ estimation on a daily basis without the use of specialized devices, e.g., spirometer. To calibrate the data from the smartphone-acquired signal, we propose using a widely-available volumetric incentive spirometer, of the sort that patients are often sent home with after hospitalization for a surgery. We further designed and implemented a smartphone application on an HTC One M8 Android smartphone, which allows recording of the chest movement signal, its calibration, and final V_T_ estimation. We tested the performance of the proposed approach by simultaneously recording a reference volume signal from a spirometer.

In agreement with our previous study [20], we found that the peak-to-peak amplitude of the smartphone-acquired chest movement signal is highly linearly correlated to tidal volume as measured by a spirometer system for twelve healthy volunteers. When the linear regression equation was used to normalize the smartphone data to tidal volume estimates, we found an RMSE of 0.147 ± 0.044 L, which corresponded to a NRMSE of 14.499% ± 4.255% when normalized to the mean value of the reference V_T_. In turn, these errors were not statistically significantly different from those found in our previous study. At this stage, the proposed method for V_T_ estimation using a smartphone’s camera has provided an average error of approximately 15% when calibrated using spirometry data as reference. However, it should be noted for normal ranges of tidal volume (~400–500 mL), the absolute error value is smaller than for the high tidal volume range (>1.5 L) as seen in Figure 4, Figure 5 and Figure 6. Note also that, as with other non-contact optical breathing monitoring methods, the calibration and tidal volume estimation results will be affected by, among other factors, the distance from and body angle of the subject with respect to the smartphone’s camera.

Regarding wearing different types of clothes, the subjects were allowed to wear any pattern, e.g., plain, dotted, stripes, and colors of their clothing during the maneuvers. Thus far, we have not noticed any significant difference in the results with different types of clothes. However, we did notice that the quality of the signal decreased when clothes with smaller dots or prints were worn. We have also performed some recordings from subjects with bare skin and we were still able to obtain good data.

A limitation of our previous study [20] was noted to be that our approach has to be calibrated using a spirometer device; this specialized device is not commonly available outside research and clinical settings. In order to deal with this calibration restriction, here we proposed to take advantage of the highly linear correlation between the chest movement signal and reference tidal volumes, to obtain a linear calibration model using only two sets of data points gathered while the volunteers breathed through an IS. The IS device is a cheap device that is currently widely used in practice in many hospitals and nursing homes for the purpose of rebuilding diaphragm muscles for those subjects who have been on a respirator or immobilized for several days due to surgery. Each data set consisted of the peak-to-peak amplitudes of the chest movement signal during inspirations at 250 and 500 mL targets marked on the IS. To minimize the effect of a possible outlier when breathing at IS targets, the median value of each data set was taken as representative to compute the calibration model. We found that when calibrated using the linear model from the first IS maneuver, the smartphone-based V_T_ estimation provided a RMSE of 0.189 ± 0.074 L equivalent to 18.559% ± 6.579% when normalized. This error represents a statistically-significant increment of around 4% compared to the NRMSE error obtained from calibration using a spirometer. Also, in contrast to the V_T_ estimation obtained from calibration via spirometer, we found a statistically-significant fixed bias of −51 mL when the calibration was performed using data from the IS maneuver. This higher estimation error and systematic V_T_ underestimation could be attributable to limitations of the IS which does not offer a more precise estimation of the inspired volume due to its coarse volume scale as well as to the increase in airway resistance when using it, which in turn increases the chest movements due to a higher breathing effort and hence shifting of the peak-to-peak of chest movement signal to higher values from which the calibration model is constructed. Some of the estimation error can be attributed to the fact that when the IS was calibrated using a calibration syringe and we found that the former is off by ~2%–3% when compared to the latter. Moreover, it should be noted that despite the best attempts by the subjects to hit the predefined target volumes, they often either under or over-achieved the target volume. Besides these performance degradations, and even when calibration should be performed *on-site* prior estimating V_T_ for a given breathing maneuver, the proposed calibration procedure is easy-to-perform and does not employ a specialized nor expensive device. The calibration procedure itself, both maneuver and calculation takes less than 30 s and is automatically performed by the smartphone app, with the option to be remotely started and completed via a wireless controller. Note, however, that the proposed methodology requires individualized calibration prior to tidal volume measurement using a smartphone camera. Hence, it is necessary for subjects to be familiar with the calibration procedure and the correct use of the IS in order to minimize estimation errors.

In this study, the deployment of an incentive spirometer made it possible to perform an individualized calibration procedure, and hence made possible the V_T_ estimation in everyday settings. Taking into account the behavior of the estimation error at the different volume levels, as shown in Figure 6, where the dispersion of the smartphone-based estimates increases at high volume levels, as well as by considering the use of the IS device to improve patients’ breathing after surgery, we envision subjects using the methodology proposed in this study at their homes to estimate the progress in their V_T_ recovery. The person would place their smartphone at a fixed location, stand still in front of it and conduct a series of breathing routines to obtain some volume estimates. By doing so, this would also minimize the motion artifacts.

Despite the easy calibration procedure proposed here, we recognize the limitations of our study. First, breathing data were collected while the healthy subjects were standing still, *i.e.*, performance of the V_T_ estimation during motion, postural changes, and airway obstruction was not explored. The second limitation of the study is the low number of subjects tested. A future study involving a larger sample size with different age categories as well as balanced gender groups will be necessary in order to make final conclusions about the suitability and performance accuracy of the proposed method. Third, only a limited area of the anterior chest wall was monitored, *i.e.*, the rig cage area using a rectangular ROI, and this could ignore small contributions of other compartments and anatomical distortions in other areas. Both limitations are topics of further exploration in our research group. In particular, we are interested in the implementation of algorithms to deal with body motion artifacts, as proposed in the literature for RR monitoring [19,25]. The implementation of image processing techniques to monitor a ROI beyond a simple rectangular area is also pending work. Note, however, that scenarios including motion artifacts are less likely to occur when measuring V_T_ during a short maneuver but it would become an important issue if continuous monitoring were intended. In addition, analyzing the performance of the smartphone-based estimator in different postures including supine, when the abdominal mechanical degree of freedom is expected to dominate the contribution to V_T_, is pending. The analysis of the performance of the proposed tidal volume estimation method at different levels of illumination, distance from and angle of the subject’s thoracic area with respect to the smartphone’s camera are other pending topics to be explored in the future. Currently, our research group is exploring other applications for the developed smartphone-based monitor in the area of respiratory sound analysis where a temporal reference would be helpful to classify and characterize the recorded sounds, particularly in patients presenting adventitious respiratory sounds.

The development of an inexpensive and portable breathing monitoring system for on-demand V_T_ and RR estimation capabilities is still pending for the general population. The near-ubiquity of smartphones and their owners’ high reliance on them makes them an attractive alternative to develop a system with those characteristics. Although several advances have been made regarding cardiac monitoring using smartphones, a limited number of studies have addressed their applications to respiratory monitoring, and most of them have focused on respiratory rate estimation despite the importance of monitoring respiratory depth. The results found in this study support the feasibility of developing a smartphone-based breathing monitor that provides V_T_ estimates when calibrated using a simple, affordable, and widely-accessible external device. Development of such a system would advance on-demand monitoring by providing another breathing parameter in addition to the number of breaths-per-minute.

## Figures and Tables

**Table 1:** Tidal volume estimation results from smartphone-acquired signals compared to the reference volume from the spirometer (*N* = 12 subjects).

Parameter		Linear Regression of Smartphone Data			Calibration of Smartphone Data Using IS		
*RMSE*	[L]	0.147	±	0.044	0.189	±	0.074
*NRMSE*	[%]	14.499	±	4.255	18.559	±	6.579

Values presented as mean ± standard deviation.
